# Supplementary material for: Sexual Assault in an Adolescent Female: A Pediatric Simulation Case for Emergency Medicine Providers
Source: MedEdPORTAL. 2020 Aug 26;16:10942. doi: 10.15766/mep_2374-8265.10942 (PMC7449576; doi:10.15766/mep_2374-8265.10942)
Supplement: Supplementary file 1 — Simulator.docxForensic Evidence Collection Primer.docxCard Layout.docxSexual Assault Case.docxCritical Actions Checklist.docxDebriefing Presentation.pptPostsession Survey.docxFollow-up Survey.docx [file mep_2374-8265.10942-s001.zip › B. Forensic Evidence Collection Primer.docx]

1. First complete the information on the outside of the kit (patient name, MRN, name of examiner and assisting nurse/tech).
2. Allow the patient to have a support person (preferably of the same gender) in the room during the examination.
3. Inform the patient what the examination entails, obtain his/her verbal and written consent; this form can be scanned into EPIC and the paper copies can go to Medical Records.
4. To label each envelope you can print patient labels from EPIC (at least 40) and then sign your name with the time on each envelope. If evidence was not collected for that particular envelope please print why the evidence was not collected (e.g. patient did not scratch alleged perpetrator).
5. Using the "State of Connecticut Sexual Assault Medical Report" form, fill in the history of assault and significant past medical history. These are the forms that have the yellow duplicate copy. Any history forms that are not used can have a slash mark through them. The patient’s ID label should be at the top of all the sheets. Complete form and put yellow copy in the envelope on the back of the kit and **seal the envelope with a patient label or tape- DO NOT LICK THE ENVELOPES.** The white copies of State of Connecticut Sexual Assault Medical Report pages 1-5 can be scanned by the IA into EPIC (can be seen in Media tab).
6. Now proceed to the physical examination and forensic evidence collection:

a. Wear a mask and try to change gloves frequently, especially if moving from one area of the body to another (e.g. finger evidence collection to GU region evidence collection). A

b. Air dry swabs as much as possible: can poke holes in bottom of paper cups labelled with the Envelope # and place wet swabs cotton tip up to air dry before placing in kit.

c. The instructions on each envelope are self-explanatory. If possible, please read before collecting evidence for that particular envelope.

**Below is a primer/summary of the steps for evidence collection for Envelopes 1-12.**

**A. Envelope 1**: One purple top tube from **Envelope 1**, as well as 2 red tops for HIV EIA, VDRL and Hepatitis panel, need to be obtained by venipuncture. **Remember that the STI testing (2 red tops) goes to the hospital laboratory and not in the Rape Kit**.

### B. Envelope 2: Obtain Oral swab and smear if there was oral-genital contact or if there are bite marks on the patient, or if the patient bit the alleged perpetrator. Swab between the buccal mucosa and mandibular and maxillary gingiva. Roll the swab onto the slide and air dry both slide and swabs and place into appropriate boxes for swabs, labelling the outside of the box which was used for slide smear.

**C. Envelope 3**: Have the patient undress over two large pieces of paper that are in the Rape Kit. Wrap the outer clothing in the top sheet and use the second sheet to wrap the wrapped clothing. Place in paper bag; staple the bag and complete the outer label.

**Note**: If there are stains on the clothing, use a piece of paper to cover the stain, then fold the clothing.

**Note:** Place underwear in a separate small white bag, placing paper over stains, label and seal the white bag, and then place in the brown paper bag with the rest of the clothes.

**D. Envelope 4: Debris collection:** Any items (e.g. hair, twigs, grass) remove from body carefully and place in envelope. Mark on diagram on other side of envelope where it was collected.

**E. Envelope 5: Swabbing of fingernails.** Use a saline moistened cotton swab to swab around cuticles.

**F. Envelope 6**: **Dried secretion specimen**: Examine the skin for any body fluids, which will appear bright yellow or orange with the Blue Maxx light (**can be found in the locked closet in the administrative office suite (1-2-3 code).** Any substance that fluoresces should be swabbed with a saline-moistened cotton swab**.** Dry the swabs as best as possible and place back in the paper packets, labeling the outside with the location of the area that was swabbed. You may need more than one cotton swab packet. All packets should be placed in Envelope 6

**G. Envelope 7: Touch DNA.** Any place on the body where the patient says the alleged perpetrator touched with fingers, swab that area with a moistened swab, air dry and place back in swab envelope. Indicate on diagram on back side of envelope where on the body this swab was obtained**.**

**Envelopes 8-12:**

**Genital swabs and samples should be collected by the** **APP, resident, fellow or attending**. Proceed to the GU examination. Note Tanner stage and the presence of any external injuries or lesions.

**H. Envelope 8**: Place the paper under the patient and **comb the perineum**, even if patient has shaved, onto the paper. Place comb inside of paper and fold up, tape to secure and place in envelope.

**I. Envelope 9**: Inspect the perineum with the Blue Maxx; any substance that fluoresces can be swabbed with a saline-moistened cotton swab, placed in its packet and then placed in an envelope or in **Envelope 9**. If no substances seen, then swab the areas between the leg and groin creases.

**J. Envelope 10 Vaginal swabs** should only be completed for **girls Tanner stage 3 or greater**. A speculum is not needed to obtain these samples, especially in adolescents who have not had a prior speculum examination. Blind vaginal swabs are adequate for the Rape Kit. Roll the swab onto the slide and air dry both slide and swabs and place into appropriate boxes for swabs, labelling the outside of the box which was used for slide smear.

K. Complete **Envelope 11** if there was a history of **anal-genital contact**. STI swabs can be taken from the anus after the swabs from the Rape Kit have been collected if needed. Roll the swab onto the slide and air dry both slide and swabs and place into appropriate boxes for swabs, labelling the outside of the box which was used for slide smear.

**L. Envelope 12 Other physical evidence.**

- 1. Any foreign bodies (tampons, condoms) found on GU examination can be placed in the plastic bags

1. Seal the kit. If detectives are not available to pick up the kit, contact the security personnel in the triage area of the Adult ED so the kit can be stored in the locked refrigerator in the AED triage security office. Any person who handles the kit needs to write their name on the chain of possession section of the outside of the kit.
2. **Please document to whom the rape kit was handed (badge number name and town of police department).**

# Evaluation for "Date Rape" Drugs

If the patient presents with altered mental status, memory loss, impaired motor skills, one should be concerned about concomitant ethanol or drug use either voluntarily by the patient or given to the patient by the perpetrator without the patient's knowledge or consent. Most often ethanol is used to facilitate a sexual assault. In rare cases, GHB or Rohypnol may be given, which are not detected by the standard toxicology screen used by the hospital. If there is a concern that drugs were given to the patient to facilitate a sexual assault, a toxicology screen (blood and urine) should be sent to the hospital laboratory. In addition, to detect GHB and Rohypnol, a separate forensic toxicology kit is available and should be kept with the Rape Kit so it can be given to detectives. Consent to obtain this testing should be documented in the EMR.

### Collection procedure

1. **Blood**: Collect 10 cc in 2 gray top tubes. Label each tube with the patient's name, date of birth, date of collection, and the initial of the person who obtained the blood. Place in the toxicology kit.
2. **Urine**: Collect 30 cc of a mid-stream urine sample into a sterile container. Label the container in the manner described above. Place in the toxicology kit and seal the kit.
3. **If no toxicology kit is available**, the collect 2 gray top tubes for blood and the urine and put these samples in a stapled biohazard bag, with a patient ID label on the front. Store the kit with the rape kit, either in the Adult ED triage security office refrigerator or hand to the detective, documenting name and badge number in the EMR.
