# Supplementary material for: Sexual Assault in an Adolescent Female: A Pediatric Simulation Case for Emergency Medicine Providers
Source: MedEdPORTAL. 2020 Aug 26;16:10942. doi: 10.15766/mep_2374-8265.10942 (PMC7449576; doi:10.15766/mep_2374-8265.10942)
Supplement: Supplementary file 1 — Simulator.docxForensic Evidence Collection Primer.docxCard Layout.docxSexual Assault Case.docxCritical Actions Checklist.docxDebriefing Presentation.pptPostsession Survey.docxFollow-up Survey.docx [file mep_2374-8265.10942-s001.zip › D. Sexual Assault Case.docx]

| Appendix D: MedEdPORTAL Simulation Case Template  SIMULATION CASE TITLE: Sexual Assault in an Adolescent Female: A Pediatric Simulation Case for Emergency Medicine Providers  AUTHORS: Kirsten Bechtel, Ambika Bhatnagar, Melissa Joseph, Marc Auerbach | |
| --- | --- |
| PATIENT NAME: Madison Rogers  PATIENT AGE: 16 y.o.  CHIEF COMPLAINT: Vomiting, ethanol use, genital pain, and suspected sexual assault. | |
|  | |
| Brief narrative description of case | *Include the presenting patient chief complaint and overall learner goals for this case*  Madison Rogers (MR) is a 16-year-old female who presents to the Emergency Department at a community hospital with suspected ethanol intoxication and sexual assault. The patient attended a party at a house of a high school classmate earlier in the evening and drank more than 4 cups of fruit punch mixed with vodka. The last event the patient remembers is awakening in a dark room with vaginal bleeding and genital/lower abdominal pain with her pants unbuttoned and her underwear missing. She is concerned that she was raped. Physical examination reveals anxious, slightly tearful patient with bruises on inner thighs and dried white, viscous fluid on the lower abdomen. There is no vaginal bleeding or GU injury. The bedside nurse has changed her clothes into a patient gown, taken her urine sample and placed an IV, obtained a blood alcohol concentration, administered antiemetics and intravenous fluids as the patient was vomiting intractably in the triage room. Additional laboratory investigations include those for pregnancy and STI’s. If asked, the patient does not want to inform her mother and is hesitant to inform the police or file a complaint. She gives consent if taken, to allow evidence collection. Learners should recognize the diagnosis of sexual assault and should explain and get consent for evidence collection. Learners should clearly explain the role of the rape kit and the involvement of the police. The patient will also agree with STI testing, antibiotic prophylaxis, and emergency contraception. The case ends after collection of forensic evidence, administration of prophylactic medications, when the hospital social worker arrives to begin a behavioral health assessment. |
| Primary Learning Objectives | *What should the learners gain in terms of knowledge and skill from this case? Use action verbs and utilize Bloom’s Taxonomy as a conceptual guide.*   1. Use open-ended, nonjudgmental questions to obtain history. 2. Understand the role of confidentiality for reproductive health concerns in adolescents. 3. Understand the role of informed consent by an adolescent for the medical and forensic evaluation after sexual assault. 4. Recognize circumstances for reporting to Law Enforcement after sexual assault. 5. Perform primary and secondary trauma survey to look for injury, including full skin exposure with appropriate patient draping. 6. Identify the health consequences of sexual assault in adolescents. 7. Perform evaluation, testing, treatment, and referrals for health consequences of sexual assault. 8. Gain competence with forensic evidence collection. |
| Critical Actions | *List which steps the participants should take to manage the simulated patient successfully. These should be listed as concrete actions that are distinct from the overall learning objectives of the case.*   1. Designate a health care provider to obtain the history and appropriate history (Menstrual; Sexual; Contraceptive; Social; Substance or HEADSS <https://depts.washington.edu/dbpeds/Screening%20Tools/HEADSS.pdf>). 2. Designate a health care provider to perform primary and secondary trauma survey to assess for physical injury. 3. Recognize the role of the victim advocate in the ED evaluation and management. 4. Recognize the role of the bedside nurse in the ED evaluation and management 5. Designate roles as to which health care provider obtains biological samples for a rape kit, and which health care provider places the samples in and seals the kit. 6. Designate a health care provider to maintain a chain of evidence. 7. Determine laboratory studies for sexually transmitted infections. 8. Determine the need for prophylactic antibiotic medication and emergency contraception. |
| Learner Preparation | *What information should the learners be given before the initiation of the case?*  Madison Rogers (MR) is a 16-year-old female who presents to the Emergency Department with suspected ethanol intoxication and sexual assault. The patient attended a party at the home of a high school classmate earlier in the evening and drank more than 4 cups of fruit punch mixed with vodka. The last event the patient remembers is awakening in a dark room with vaginal bleeding and pain, and her pants unbuttoned and her underwear missing. She told her 17-year-old friend, who then drove her to the ED and dropped her off. |

| Initial Presentation | | | |
| --- | --- | --- | --- |
| Initial vital signs | HR: 85  ECG Rhythm: NSR  RR: 18  BP: 100/70  Sat: 99%  Temp: 37.2 | | |
| Laboratory studies | Urine toxicology: Negative  Urinalysis: Trace blood, specific gravity 1.010  Urine HCG: Negative  Blood Alcohol Concentration: 0.07% | | |
| Overall Appearance | *What do learners see when they first enter the room?*  A female adolescent mannequin in a hospital gown with the bedside nurse played by an actor. Forensic evidence kit set up on a counter with all the envelopes (1-12) with accompanying index cards with simplified steps. (Script of the history of assault provided by simulationist via microphone). The patient is on a CR monitor and has an IV placed in AC fossa because she was vomiting profusely in triage. Her vital signs (Heart rate, respiratory rate, and blood pressure) are within the normal range for a healthy 16-year-old female. | | |
| Actors and roles in the room at case start | *Who is present at the beginning, and what is their role? Who may play them?*  The patient is high fidelity mannequin; bedside nurse is a scripted actor. If a third actor is available, he/she can portray the patient advocate from the rape crisis center. | | |
| HPI | *Please specify what info here and below must be asked vs. what is volunteered by the patient or other participants.*  The patient will provide the following history unsolicited:  Was in good state of health before attending the party. Vomiting and dizzy and felt hot after drinking punch with vodka. Went to lie down in a bedroom and woke up with vaginal pain and bleeding, pain when she tried to urinate. Started vomiting in triage. The nurse placed IV, administered IVF and antiemetics, collected her clothes, and changed the patient into a gown, obtained a dirty catch urine sample and blood alcohol concentration (BAC). The nurse called the local Sexual Assault Crisis Services, and an advocate will soon be arriving at the ED.  ROS Vaginal pain and bleeding; pain with urination | | |
| Past Medical/Surgical History | Medications | Allergies | Family History |
| Has exercise-induced asthma. Last menstrual period was two weeks ago. Is not on any hormonal contraception. No prior sexual activity.  No history of behavioral health concerns. No history of substance use or misuse. | Albuterol MDI before playing ice hockey | None | Lives with mother and 21-year-old sister. |
| Physical Examination | | | |
| General | Appears uncomfortable, slightly anxious, answers questions, oriented to person, place, and time. | | |
| HEENT | normal | | |
| Neck | normal | | |
| Lungs | Clear to auscultation bilaterally | | |
| Cardiovascular | Regular in rate and rhythm, no murmurs/rubs/gallops | | |
| Abdomen | Mild diffuse tenderness, soft, nondistended | | |
| Neurological | A&Ox4, strength intact and symmetric in upper and lower extremities | | |
| Skin | Red, purple ecchymoses on interior aspects of bilateral upper thighs | | |
| GU | If a pelvic exam is performed, there is white viscous material on the lower abdomen | | |
| Psychiatric | Awake, alert, slightly anxious | | |

| Instructor Notes - Changes and CASE Branch Points  *This section should be a list with detailed description of each step than may happen during the case. If medications are given, what is the response? Do changes occur at certain time points? Should the nurse or other participant prompt the learners at given points? Should new actors or participants enter, and when? Are there specific things the patient will say or do at given times? There are a few examples given, but it is expected that most cases will have many more changes and potential branch points..* | | |
| --- | --- | --- |
| Intervention / Time point | Change in Case | Additional Information |
| When learners enter the examination room | The patient asks, "Will you have to call my mom?" | The patient tearfully states, "Please do not call my mom." |
| If the learner asks, “Why don’t you want to call your mom?” | The patient states, " She will be really mad I was drinking." | The patient asks, "Can you call my sister instead? She is 21 and can come to the ED." |
| If the learner says, "We have to call your mom." | She will respond, "Okay then, I will go home. My mom cannot know." | The nurse will say, "She is 16, she can seek care for rape without her mother knowing” (in Connecticut, this is the case; however, responses can be modified based on local consent laws for minors for reproductive health concerns). If there is a second actor available to play the advocate from the rape crisis center, scripting that reflects this actor can use the local laws and guidelines for adolescents and consent for evaluation for sexual assault examination instead of the bedside nurse. |
| If the learner says, "It is okay; we do not have to call your mom." | The patient will say, "She will get mad I was drinking. I am ashamed this happened to me." | She will respond, "I can tell my sister, and she can help me tell my mom."  If a second actor is available to play the advocate from the rape crisis center, scripting that reflects the local laws and guidelines for adolescents and consent for evaluation for sexual assault examination can be used. |
| The learner explains to the patient that a rape kit can be collected to see if there is any evidence on her body that could identify who might have assaulted her. | The patient agrees to have a rape kit done, but then asks, "I do not want to make a police report, everyone will know at school and say I am a slut even though I am a virgin.” | The nurse (or the advocate from the rape crisis center) can explain that an anonymous rape kit can be collected, where the patient’s identity is not revealed unless she decides to make a report to the police (This is local Connecticut state statute; scripting that reflects the local laws and guidelines for anonymous forensic evidence collection for sexual assault examination can be used). |
| If the learners look carefully at the lower abdomen, they may note white, viscous fluid over the lower abdomen. | If learner asks the patient about the substance, she will say, "I do not know what that is, it was in my underwear too. Maybe that is what I thought was blood.” | The nurse will say, “You can swab that with a cotton swab and use it for the sample for Envelope 6: Dried secretion specimen.” |
| During the examination of the GU region, the learners see an otherwise normal GU examination of a 16-year-old female (can provide a slide of a Tanner 5 female GU anatomy that is normal if available, otherwise note that her GU examination is normal for age), without any blood noted from the vaginal vestibule. | If the learners want to perform a speculum examination, the patient becomes anxious and says she does not want any further examination of her GU area, including any swabs for the rape kit | If the learners try to persuade the patient to continue with the GU examination, the bedside nurse will state, “She can refuse any part of the examination, you should not force her to be examined.” |
| During the examination of the skin, if the learners expose the upper thighs, they will see red, purple ecchymoses on interior aspects of bilateral upper thighs. |  | The nurse will prompt the learners that swabs of the bruises can be used for Envelope 7: Touch DNA, where an alleged perpetrator's fingers may have contacted the patient's skin if this DNA technology is not available based on local law enforcement protocols for forensic evidence collection. The bedside nurse will also recommend documenting the location and appearance of the bruises in the medical record. |

See Appendix A.

Ideal Scenario Flow

*Provide a detailed narrative description of the way this case should flow if participants perform in the typical fashion.*

*Include the presenting patient chief complaint and overall learner goals for this case*

Madison Rogers (MR) is a 16-year-old female who presents to the Emergency Department at a community hospital with suspected sexual assault. The patient attended a party at a house of a high school classmate earlier in the evening and drank more than 4 cups of fruit punch mixed with vodka. The last event the patient remembers is awakening in a dark room with vaginal bleeding and genital/lower abdominal pain with her pants unbuttoned and her underwear missing. She is concerned that she was raped. Physical examination reveals anxious, slightly tearful patient with bruises on inner thighs and dried white, viscous fluid on the lower abdomen. There is no vaginal bleeding or GU injury. The bedside nurse has changed her clothes into a patient gown, taken her urine sample and placed an IV, obtained a blood alcohol concentration, administered antiemetics and intravenous fluids as the patient was vomiting intractably in the triage room. Additional laboratory investigations include those for pregnancy and STI’s. If asked, the patient does not want to inform her mother and is hesitant to inform the police or file a complaint. She gives consent to allow evidence collection, but does not want to notify the police or her mother; instead, she wants to call her adult sister for support. The patient will also agree with STI testing and emergency contraception. The case ends after collection of forensic evidence, administration of prophylactic medications, when the hospital social worker arrives to begin a behavioral health assessment.

*Provide a list of management errors or difficulties that are commonly encountered when using this simulation case.*

1. *Learners do not fully expose the patient's skin and fail to see the dried viscous fluid on the lower abdomen and the bruising on the bilateral inner thighs: We found that when first developing this simulation with EM trainees that they did not fully expose the skin when examining the patient so as not to upset the patient. We modified our sessions to include feedback regarding draping for the patient’s comfort and an explanation of why it was essential to perform a full skin examination to ensure no injuries or evidence of sexual assault was missed.*
2. *Learners insist that the patient’s mother be notified: Some of our learners insisted that the patient’s mother be notified as she was 16 years old. In Connecticut, a 16-year-old patient can consent for medical and forensic evaluation for sexual assault without a guardian's permission. However, responses can be modified based on local consent laws for minors for reproductive health concerns and evaluation for sexual assault.*
3. *Learners will attempt to perform a speculum examination of the cervix in a patient who has not previously been sexually active and who becomes anxious when this is attempted: A few of our learners believed that the source of possible vaginal bleeding should be sought by doing a speculum examination. However, because the patient has no signs of external GU injury or bleeding at the vaginal vestibule, this can be deferred so as not to make the patient more anxious and at her request.*
