# Supplementary material for: Sexual Assault in an Adolescent Female: A Pediatric Simulation Case for Emergency Medicine Providers
Source: MedEdPORTAL. 2020 Aug 26;16:10942. doi: 10.15766/mep_2374-8265.10942 (PMC7449576; doi:10.15766/mep_2374-8265.10942)
Supplement: Supplementary file 1 — Simulator.docxForensic Evidence Collection Primer.docxCard Layout.docxSexual Assault Case.docxCritical Actions Checklist.docxDebriefing Presentation.pptPostsession Survey.docxFollow-up Survey.docx [file mep_2374-8265.10942-s001.zip › E. Critical Actions Checklist.docx]

Appendix E. Critical Actions Checklist Critical Actions (Clinical Management):

Use open ended, nonjudgmental questions to obtain history.

Explain limits of confidentiality for reproductive health and substance use concerns.

Perform primary and secondary trauma survey to look for injury.

Intervene to identify and prevent medical consequences of sexual assault.

Intervene to evaluate patient for behavioral health consequences of sexual assault.

Demonstrate the collection of forensic evidence.

Demonstrate knowledge of the circumstances for reporting to Law Enforcement.

Critical Actions (Teamwork and Communication):

Designate a health care provider to obtain the history.

Designate a health care provider to perform primary and secondary trauma survey to assess for physical injury.

Employ the victim advocate to support the patient during the ED evaluation and management.

Employ the social worker to evaluate the patient for behavioral health sequelae of sexual assault.

Designate roles as to which health care provider obtains biological samples for rape kit, and which health care provider places the samples in and seals the kit.

Designate a health care provider to maintain chain of evidence.

Pre-­‐procedure

 Pre-huddle by participants to assign roles for patient evaluation (history, PE, collection of evidence)

Introduces members of health care team and roles to the patient.

 Verbalizes indications for procedure (forensic evidence kit).

 Verbalizes appropriate informed consent for procedure (forensic evidence kit).

 Verbalizes indications for collection of an “anonymous” kit.

Patient history

 Obtains history of assault (e.g. time, contact, patient actions afterward).

 Obtains relevant past medical history.

 Obtains relevant reproductive health history.

 Obtains relevant behavioral health history.

Patient preparation

 Assesses for competency in consenting/assenting to examination and evidence collection (e.g. notification of parents/guardians).

 Allows for parent proxy for support (family member, rape crisis center worker).

 Allows for patient preferences for order of examination.

 Explains risks and benefits of evidence collection (e.g. Discusses role of law enforcement in forensic evidence collection).

 Allows for patient questions regarding examination and evidence collection.

Patient examination

 Performs primary survey for trauma (general).

 Performs secondary survey for trauma (specific to sexual assault).

Performs evidence collection

 Collects clothes.

 Collects oral swabs.

 Collects hand swabs.

 Collects GU swabs.

 Collects secretion swabs.

 Dons mask and changes gloves in between steps (verbalizes).

 Seals kit.

Post procedure

 Discusses health consequences of sexual assault (e.g. STI, Pregnancy, behavioral health).

 Discusses treatment for health consequences of sexual assault (e.g. prophylaxis).

 Discusses behavioral health evaluation (e.g. SW evaluation).

 Discusses follow up planning post ED discharge (e.g. where to get test results).
