# Supplementary material for: Sexual Assault in an Adolescent Female: A Pediatric Simulation Case for Emergency Medicine Providers
Source: MedEdPORTAL. 2020 Aug 26;16:10942. doi: 10.15766/mep_2374-8265.10942 (PMC7449576; doi:10.15766/mep_2374-8265.10942)
Supplement: Supplementary file 1 — Simulator.docxForensic Evidence Collection Primer.docxCard Layout.docxSexual Assault Case.docxCritical Actions Checklist.docxDebriefing Presentation.pptPostsession Survey.docxFollow-up Survey.docx [file mep_2374-8265.10942-s001.zip › G. Postsession Survey.docx]

**Sexual Assault Forensic Examination Training**

Q0. Thank you for participating in today's session. The goal of this training is to enhance providers' knowledge, skills and attitudes related to the care of patients with sexual assault.

Q1a. Did you review any of the materials emailed to you before the simulation (e.g., Primer)?

- Yes
- No
- I didn't receive any materials

Q1. Please estimate the number of sexual assault patients you have treated in the past year. (list ZERO IF NONE)

Click to write number [ ]

Q2. Please estimate the number of sexual assault patients you have performed a “kit” on in the past year. (list ZERO IF NONE)

Click to write number [ ]

Q3. Please estimate the number of hours of prior training in treating patients presenting after sexual assault. (list ZERO IF NONE)

Click to write number [ ]

Q4. I encounter the following BARRIERS to treating a sexual assault victim (Click all that apply)

- Time
- Training
- Equipment
- Personal discomfort
- Inadequate Space
- Other: [ ]

Q5. BEFORE this training session I was comfortable/confident treating a patient presenting after sexual assault.

- Strongly agree
- Agree
- Somewhat agree
- Neither agree or disagree
- Somewhat disagree
- Disagree
- Strongly disagree

Q6. AFTER this training session I am comfortable/confident treating a patient presenting after sexual assault.

- Strongly agree
- Agree
- Somewhat agree
- Neither agree or disagree
- Somewhat disagree
- Disagree
- Strongly disagree

Q7. Please check all of the objectives that you achieved during this training session. Please check more than one if appropriate:

- HISTORY: I can elicit a history using patient centered and sensitive language from a patient presenting after a sexual assault.
- KIT: I can independently demonstrate all of the steps required for the collection of evidence using the “kit” for a patient presenting after a sexual assault.
- TREATMENT/FOLLOW-UP: I can describe the health consequences and follow-up plan to a patient presenting after a sexual assault.
- TREATMENT/FOLLOW-UP: I can describe the medication treatment options to a patient presenting after a sexual assault
- LEGAL: I can describe the mandated reporting laws and state requirements for evidence collection for patients presenting after a sexual assault in CT

Q8. How can we improve this training?

Q9. What part of this training was most valuable to you?

Q10. If you have any other comments on this training, please enter them below:
