# Supplementary material for: Sexual Assault in an Adolescent Female: A Pediatric Simulation Case for Emergency Medicine Providers
Source: MedEdPORTAL. 2020 Aug 26;16:10942. doi: 10.15766/mep_2374-8265.10942 (PMC7449576; doi:10.15766/mep_2374-8265.10942)
Supplement: Supplementary file 1 — Simulator.docxForensic Evidence Collection Primer.docxCard Layout.docxSexual Assault Case.docxCritical Actions Checklist.docxDebriefing Presentation.pptPostsession Survey.docxFollow-up Survey.docx [file mep_2374-8265.10942-s001.zip › H. Follow-up Survey.docx]

Sexual Assault Forensic Examination Training Follow-Up Survey

Q1. Postgraduate year of training

- PGY 1
- PGY 2
- PGY 3
- PGY 4

Q2. I am confident treating a patient presenting after sexual assault.

- Strongly agree
- Agree
- Somewhat agree
- Neither agree nor disagree
- Somewhat disagree
- Disagree
- Strongly disagree

Q3. For a patient with sexual assault, I am comfortable in managing the following:

- HISTORY: I can elicit a history using patient centered and sensitive language from a patient presenting after a sexual assault.
- KIT: I can independently demonstrate all of the steps required for the collection of evidence using the “kit” for a patient presenting after a sexual assault.
- TREATMENT/FOLLOW-UP: I can describe the health consequences and follow-up plan to a patient presenting after a sexual assault.
- TREATMENT/FOLLOW-UP: I can describe the medication treatment options to a patient presenting after a sexual assault
- LEGAL: I can describe the mandated reporting laws and state requirements for evidence collection for patients presenting after a sexual assault in CT

Q4. Since participating in the Sexual Assault Simulation, have you treated a patient with sexual assault?

- Yes
- No 🡪 If so, skip to Q8

Q5. If yes, how many patients with a history of sexual assault have you evaluated and/or managed?

- Male patients with sexual assault [ ]
- Female patients with sexual assault [ ]
- Total [ ]

Q6. Was there any component of the simulation that you found helpful In your subsequent care of sexual assault patients?

- Yes
- No 🡪 If so, skip to Q8

Q7. If yes, what component most helpful? (can choose more than one answer)

- History and physical exam simulation
- Availability of forensic evidence kit during the simulation
- Performance of the forensic kit steps
- Prompting cards for each step of the kit
- Availability of other team members during the case
- Debriefing session
- Other

Q8. Would you recommend the Sexual Assault Simulation training to other trainees?

- Yes
- No

Q9. If you have any other comments about your participation in the Sexual Assault simulation or the care of Sexual assault patients, please enter them below.
