# Supplementary material for: Sexual Assault in an Adolescent Female: A Pediatric Simulation Case for Emergency Medicine Providers
Source: MedEdPORTAL. 2020 Aug 26;16:10942. doi: 10.15766/mep_2374-8265.10942 (PMC7449576; doi:10.15766/mep_2374-8265.10942)
Supplement: Supplementary file 1 — Simulator.docxForensic Evidence Collection Primer.docxCard Layout.docxSexual Assault Case.docxCritical Actions Checklist.docxDebriefing Presentation.pptPostsession Survey.docxFollow-up Survey.docx [file mep_2374-8265.10942-s001.zip › _Educational_Summary_Report_10942.pdf]

MedEdPORTAL® The AAMC Journal of Teaching and Learning Resources

Original Publication

Open Access

Sexual Assault in an Adolescent Female: A Pediatric Simulation Case for Emergency Medicine Providers

Kirsten Bechtel, MD*, Ambika Bhatnagar, MBBS, Melissa Joseph, MD, Marc Auerbach, MD

*Corresponding author: kirsten.bechtel@yale.edu

Abstract

Introduction: Many emergency medicine (EM) physicians have limited training in the care of sexual assault patients. Simulation is an effective means to increase the confidence and knowledge of physicians in such high-stakes, low-frequency clinical scenarios as sexual assault. We sought to develop and implement a sexual assault simulation with a structured debriefing for EM residents and to determine its impact on resident learners' attitudes and knowledge skills in the care of patients with sexual assault. **Methods:** The simulation blended psychomotor skills (e.g., collecting forensic evidence), cognitive skills (e.g., ordering laboratory studies and medications), and communication skills (e.g., obtaining relevant patient history, responding to psychosocial concerns raised by team members and simulator). Our emergency department checklist was available as a cognitive aid for each step of the evidence collection process. A content expert answered questions in real time during the simulation and provided structured debriefing following the simulation. Trainees completed an anonymous survey within a week after the intervention and a follow-up survey within 8 months. **Results:** Nineteen EM trainees participated. Presimulation, 39% reported never having received training in the medical care of a patient with sexual assault. The proportion of trainees agreeing or strongly agreeing with the statement "I am comfortable and confident managing a case of sexual assault" increased from 21% to 74% following the simulation ($p < .05$). **Discussion:** This intervention was associated with EM trainees' increased confidence with and knowledge of medical and forensic evaluations for an adolescent with sexual assault.

Keywords
Sexual Assault, Adolescent, Sexual Abuse of Child, Consent, Forensic Evidence Collection, Emergency Medicine, Case-Based Learning, Pediatric Emergency Medicine, Simulation

Educational Objectives

By the end of this activity, learners will be able to:

1. Employ patient-centered and sensitive language when eliciting a history from an adolescent with sexual assault.
2. Identify medical and behavioral health consequences of sexual assault in adolescents.
3. Demonstrate greater competence and confidence in collecting evidence for the rape kit.

Introduction

Sexual assault nurse examiner (SANE) programs were developed in 1976 to improve forensic evidence collection and delivery of care to victims of sexual assault. Today, with over 800 programs across the nation, SANE nurses are currently the primary health care providers managing patients with sexual assault and completing sexual assault forensic evaluation (SAFE) kits or rape kits.[1] In an emergency department (ED) with a SANE program, a trained nurse is available 24 hours a day to provide adequate and timely care for victims of sexual assault who present to the ED.[2] The presence of a SANE program has been shown to enable comprehensive management, complete evidence collection, healthy relationship development, better psychosocial recovery, and follow-up care among patients with sexual assault.[2-7] In contrast, institutions without SANE programs have been shown to have frequently incomplete SAFE kits,[8] missed emergency contraception[9] and sexually transmitted infection screening, incomplete documentation, and lack of appropriate referrals.[10]

Despite this improved delivery of care, less than one-fifth of US hospitals with emergency rooms have all of the services required for comprehensive medical care of patients with sexual assault.[11,12] This has resulted in a disparity in the quality of care

Citation:
Bechtel K, Bhatnagar A, Joseph M, Auerbach M. Sexual assault in an adolescent female: a pediatric simulation case for emergency medicine providers. *MedEdPORTAL*. 2020;16:10942.
https://doi.org/10.15766/mep_2374-8265.10942

Copyright © 2020 Bechtel et al. This is an open-access publication distributed under the terms of the Creative Commons Attribution license.

delivered to patients with sexual assault across the US health system. Campbell and Bybee described this disparity in health services from a patient's perspective by interviewing 147 victims of sexual assault.[13] They found that not having a SANE program was among the factors that negatively impacted the management of a patient.

Specialized teams, including those with SANEs, can improve care.[10,14-16] However, in community hospitals, training forensic nurses and maintaining their competency are often limited by short staffing and lack of funding.[17] If such teams are not feasible, specific clinical pathways may provide an alternative way to improve the medical care of adolescents with sexual assault.[18] However, for sexual assault victims who seek forensic evaluation, there exists no similar specialized clinical pathway to assist ED providers in the collection of forensic evidence.

Additionally, most physicians have limited training in forensic evidence collection either due to the infrequency of the event or because of the predominance of SANE programs in academic medical centers where most physicians are trained.[19] Multiple national studies have shown that the majority of sexual assault victims are young females, with 42% of female rape victims being less than 18 years of age.[20] Most of these adolescent victims are seen in community EDs near to the place where they live or where the assault was committed. Training forensic nurses and maintaining their competency in these small community hospitals are unattainable due to funding constraints and short staff composition.[17]

To bridge the gap in knowledge and skills among non-SANE providers, several investigators have focused on the medical, but not the forensic, evaluation of sexual assault. Sheets and Bretl developed the SA Pocket Tool to help support providers who evaluate children for sexual abuse.[21] Their tool was not, however, designed to be used as a checklist or as a stand-alone how-to manual for forensic evidence collection. It also was not formally evaluated. Similarly, Siegel and colleages developed on-the-go downloadable videos of the medical evaluation and treatment of adult sexual assault patients for medical students.[22] While knowledge scores improved after viewing the videos, there was no content regarding the forensic evaluation of these patients.

Although high-fidelity simulation-based training interventions have been used to maintain competencies among SANE nurses,[17,23] the previous studies of training interventions targeted to ED residents/physicians have been low fidelity.[23-26] McLaughlin, Monahan, Doezema, and Crandall described the effectiveness of implementing an 8-hour-long training program for EM trainees, which included a combination of lectures, role-plays, and skill laboratories based on national guidelines for managing patients with sexual assault, including forensic evaluations.[24] They were able to show significant improvement in a written knowledge test and low-fidelity simulation-based evidence collection after the training intervention. Participants also had improved confidence and were satisfied with their participation in the intervention. These results were replicated by Auten and colleagues, who used a similar low-fidelity simulation-based educational intervention to train ED residents.[25] While the above interventions showed improvement in learner knowledge and skill, the investigators also incorporated an 8-hour didactic lecture[25]; such a didactic lecture may not be feasible in all EM residency training programs.

Methods

We designed this simulation-based training intervention for a small group of up to four emergency medicine trainees. Its duration was approximately 70 minutes, divided into a 5-minute prebriefing to establish expectations, confidentiality, and roles of the learners in the simulation; a 40-minute simulation exercise with a content expert available to answer learners' questions in real time; and a 25-minute structured debriefing with a content expert. Before the simulation, learners received by email an electronic document containing the ED forensic evidence guidelines, which were also made available as a cognitive aid for each step of the forensic evidence collection process during the simulation.

Development of This Intervention

We assembled a multidisciplinary team of experts to iteratively develop this simulation with the aim of participants achieving the following learning objectives:

1. Employ patient-centered and sensitive language when eliciting a history from an adolescent with sexual assault.
2. Identify medical and behavioral health consequences of sexual assault in adolescents.
3. Demonstrate greater competence and confidence in collecting evidence for the rape kit.

Our team consisted of a pediatric emergency physician with expertise in child abuse/sexual abuse, an emergency physician simulation fellow, a simulation faculty member, and a recent medical school graduate. A SANE nurse and a patient advocate from the local sexual assault crisis center also provided feedback on the content of the simulated session. The group met in person and then corresponded over email to iteratively update the content after each session.

Copyright © 2020 Bechtel et al. This is an open-access publication distributed under the terms of the Creative Commons Attribution license.

When the learners entered the simulation suite, they were introduced to Madison Rogers, a 16-year-old girl who was concerned she had been raped after passing out from alcohol intoxication while attending a party hosted by a classmate. A simulator (Laerdal SimMan with a pelvic anatomy attachment; Appendix A) served as the patient. She was vomiting and had vaginal bleeding and pain. An actor served as the bedside nurse, who placed the patient in a hospital gown, collected the patient's clothes, placed an IV, administered a normal saline bolus and ondansetron, and obtained a blood sample for the forensic kit. The nurse was available to assist the learners at any point during the simulation. The learners obtained a relevant medical history, performed a physical examination, ordered laboratory testing and postexposure prophylaxis, and collected forensic evidence from the simulator.

We used the checklist utilized by the SAFE program in our ED for the collection of forensic evidence as a cognitive aid for each step of the collection process (Appendix B). This checklist was developed to address the medicolegal aspects of the care of sexual assault patients at our institution. To facilitate learners' abilities to collect the evidence in a stepwise fashion, each envelope from the kit for each step of the collection of evidence was laid out on a counter with the required supplies for that step (saline, cups for drying specimens). Also, an index card with relevant information from the ED checklist for that step of the evidence was placed next to each envelope so learners could refer to it as they began that particular step of the forensic evidence collection (Appendix C).

During the simulation, the content expert faculty facilitator was available to answer questions in real time (reflection in action). The simulation ended when the learners completed the forensic and medical evaluations and were ready to allow a social worker to come into the examination room to perform a behavioral health assessment of the patient.

After the simulation, the faculty facilitator used the simulation guides (Appendix D) and critical actions checklist (Appendix E) with complete simulation details, as well as the debriefing materials (Appendix F), to guide learners through the simulation and debriefing discussion. The structured debriefing (reflection on action) involved reactions, analysis, and summary phases (Figure). Trainees completed an anonymous survey (Appendix G) within 1 week of the intervention and then a follow-up survey (Appendix H) within 8 months of the simulation to evaluate the simulation's impact on their subsequent care of sexual assault patients. The survey was anonymous so that participants would feel comfortable providing frank and meaningful feedback that would be used to revise and improve the simulation for future learners iteratively.

Equipment/Environment

This simulation was completed with a high-fidelity simulator with a female pelvic anatomy attachment (Laerdal SimMan with a pelvic

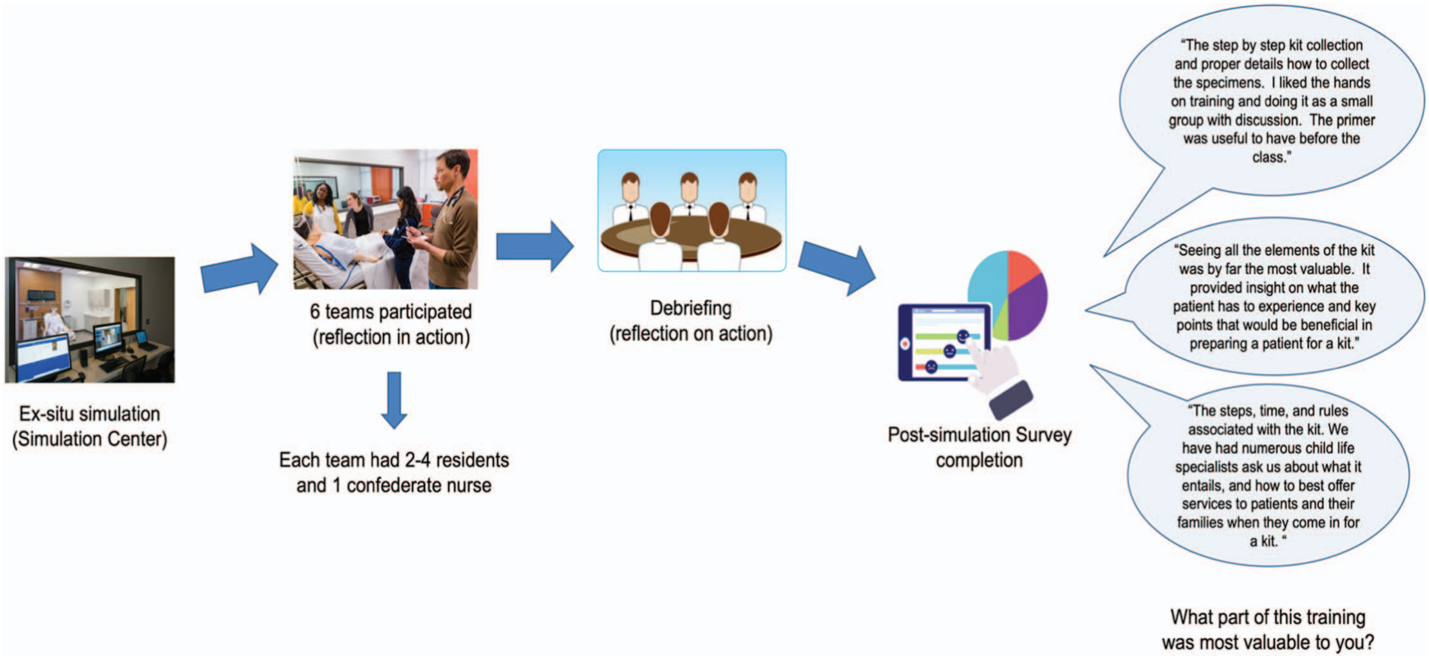

Figure. Flow diagram of simulation. Images used with permission from the Yale Medicine Simulation Center.

Copyright © 2020 Bechtel et al. This is an open-access publication distributed under the terms of the Creative Commons Attribution license.

anatomy attachment; Appendix A). However, one could use a human actor to serve as the patient, or a low-fi simulator with the anatomic model, if high fidelity is not available.

The simulator was dressed in a hospital gown and placed supine on a hospital stretcher. An IV was placed in the antecubital fossa and attached to a cardiorespiratory monitor with a blood pressure cuff. The simulator had the following moulage: red/blue bruises on the inner proximal aspects of both thighs and a white viscous substance (petroleum jelly) to represent seminal products over the suprapubic region of the lower abdomen. Other supplies included the following:

- Forensic evidence collection kit (our scenario utilized the State of Connecticut Sexual Assault Forensic Evidence Collection Kit CT-100).
- Alternate light source (Sirchie Blue Maxx) to assist learners in evaluating for possible biological secretions on the simulator that could be obtained for forensic evidence collection.
- Additional cotton-tipped swabs to use for additional specimen swabs.
- Paper cups to facilitate air drying of specimen swabs.
- Saline for moistening specimen swabs, if indicated.
- A stethoscope and otoscope to facilitate a primary and secondary physical examination of the patient for traumatic injury.
- Bedsheets to facilitate draping of the simulator during the physical examination.

Personnel

The simulation required at least three people. The first was a scripted actor who served as the bedside nurse and could be played by other trainees, faculty, or staff not participating as learners. The second was a faculty member with content expertise in the evaluation of sexual assault who facilitated the simulation and debriefing. A third person served as the simulation technologist to manipulate the vital signs and telemetry monitoring and to act as the voice of the simulator—that is, to provide verbal responses from the simulator when prompted by the learners.

When learners entered the examination room, the nurse gave a report on the patient based on the scripted scenario, assisted in carrying out orders, and prompted learners with information as needed based on the scenario flow and desired responses. The patient simulator, via the simulation technologist, provided the appropriate history and examination responses when prompted by the learners. Scripting of the responses of the nurse and patient simulator provided a standardized interaction for learners. A faculty facilitator with content expertise in sexual assault answered any of the learners' questions during the simulation and guided the debriefing to ensure that the relevant cognitive, technical, and behavioral learning objectives were achieved.

Assessment

The faculty facilitator provided a detailed formative assessment using the critical actions checklist (Appendix E). Items for the critical actions checklist were created based on the existing published clinical guidelines for the management of adolescent sexual assault[27] and the expertise of the faculty facilitator. The checklist was iteratively revised to include items identified as important, necessary, and within the scope of knowledge for emergency medicine trainees.

Debriefing

The faculty facilitator with expertise in the management of acute sexual assault provided formative assessment using direct observation and real-time feedback during the simulation. In the first series of simulations, additional content experts (a SANE nurse and a patient advocate from the local sexual assault crisis center) attended to provide feedback and contribute to debriefing. We informed the participants at the beginning of simulation that their performance in the simulation would not be used as a competency evaluation and would not be included in their formal evaluations. The understanding that simulation was a safe space to make mistakes and ask questions was distinctly emphasized in the prebriefing discussion before the start of the simulation. The critical actions checklist (Appendix E) was developed as a guide for discussion and reflection as opposed to a performance assessment tool.

The small-group debriefing session occurred immediately following the completion of the simulation. The faculty facilitator began by asking the learners to reflect on their simulation experience. Each learner was offered a chance to reflect on both individual and team performance, concentrating on what was done expertly and what could be done differently. Following self-reflection, the faculty facilitator began the discovery phase by reviewing simulation events using the critical actions checklist (Appendix E) as a guide. Also, the faculty member used a PowerPoint presentation (Appendix F) to emphasize specific teaching points, such as the use of patient-centered and sensitive language when eliciting a history and obtaining consent; the medical and behavioral health consequences, evaluation, and treatment of sexual assault; and the procedures for evidence collection.

Copyright © 2020 Bechtel et al. This is an open-access publication distributed under the terms of the Creative Commons Attribution license.

After the debriefing session, learners were asked to summarize one crucial learning point that they would take away from the simulation session. The faculty facilitator also reviewed the primary learning objectives to reinforce new information or mental models learned during the simulation and related this information to a future clinical encounter with patients with sexual assault.

Results

Nineteen emergency medicine trainees participated in the simulation; demographics are in Table 1. Before participating in the simulation, 63% (12) reported having never received prior training in the medical care of a patient with sexual assault. Postsimulation, 83% (16) trainees agreed with the statement "I can elicit the relevant history from a patient with sexual assault"; 83% (16) agreed with the statement "I can independently complete the steps for the collection of forensic evidence"; 78% (15) agreed with the statement "I can describe the health consequences of sexual assault"; and 83% (16) agreed with the statement "I can describe the medical treatment options for patients with sexual assault." The proportion of trainees who agreed or strongly agreed with the statement "I am comfortable and confident managing a case of sexual assault" increased from 21% (four trainees) before the simulation to 74% (14) following it ($p < .05$; Table 2).

Trainees were surveyed anonymously within 8 months of participating in the simulation to assess its impact on subsequent care of sexual assault patients; 58% (11) responded. Of these, 72% (eight) agreed with the statement "I am comfortable and confident managing a case of sexual assault"; 81% (nine) agreed with the statement "I can elicit the relevant history from a patient with sexual assault"; and 72% (eight) said they could independently carry out the steps for forensic evidence collection. However, only 36% (four) could still describe the health consequences of sexual assault, and only 45% (five) said they could still describe the medication treatment options for patients with sexual assault (Table 2). Finally, 72% (eight) would recommend the simulation to other trainees.

Table 1. Participant Demographics ($N = 19$)

Demographic	No. (%)
Year of training	
EM 1	4 (21%)
EM 2	5 (36%)
EM 3	6 (32%)
EM 4	4 (21%)
No. of prior sexual assault patients	
0	4 (21%)
1	6 (32%)
2	2 (10%)
3	1 (5%)
4 or more	6 (32%)
No. of prior kits	
0	15 (79%)
1	3 (16%)
2	1 (5%)
Previous training on care of sexual assault cases	7 (39%)

Table 2. Survey of Beliefs Regarding Sexual Assault, Immediately Postsimulation and Follow-up

	No. (%)	
Item	Postsimulation Survey[a]	Follow-up Survey[b]
I can elicit the relevant history from a patient with sexual assault.	16 (83%)	9 (81%)
I can independently complete the steps for the collection of forensic evidence.	16 (83%)	8 (72%)
I can describe the health consequences of sexual assault.	15 (78%)	5 (45%)
I can describe the medication treatment options for patients with sexual assault.	16 (83%)	8 (72%)
I am comfortable and confident managing a case of sexual assault.	14 (74%)	8 (72%)

[a]Administered within 1 week of simulation ($N = 19$).
[b]Administered within 8 months of simulation ($N = 11$).

Discussion

To address gaps in the knowledge, confidence, and competence of emergency medicine trainees in the care of sexual assault patients, we developed a high-fidelity, single-session simulation facilitated by a content expert in the evaluation of sexual assault. This simulated scenario was associated with increased confidence and perceived competence of emergency medicine trainees for performing the appropriate medical and forensic evaluations in an adolescent in an ED setting. It is evident that such training would be helpful and is necessary for emergency medicine trainees due to the dearth of SANE resources in community EDs in the US where most sexual assault victims seek care. Thus, it would be useful to fill in the gaps for both the medical and the forensic care of these primarily female adolescent patients when they seek care in the ED after sexual assault.

We have demonstrated that emergency medicine trainee confidence and perceived competence in the medical and forensic evaluation of an adolescent with sexual assault increased immediately after this single-session, high-fidelity simulation facilitated by a content expert in sexual assault. There are several advantages to this approach. First, the training can be completed in about 70 minutes, which increases the likelihood of a trainee being able to complete the entire training in a single session. The fact that this training can be completed in a single session enhances its generalizability to other emergency

Copyright © 2020 Bechtel et al. This is an open-access publication distributed under the terms of the Creative Commons Attribution license.

medicine training programs. Second, this simulation session can be adapted to the local needs of a particular training program with regard to the procedures for collecting forensic evidence within the local jurisdiction. If a training program has guidelines for the collection of forensic evidence in its ED, they can be broken down into a step-by-step fashion on index cards. They can be displayed with the materials for evidence collection in a stepwise fashion, to facilitate the learner's familiarity and knowledge with each step of the evidence collection process. Third, since there are published national guidelines on the best practices for the medical and behavioral health evaluation of adolescents with sexual assault,[27] that information is easily generalizable and can be adapted to the educational needs of an emergency medicine training program and incorporated into clinical pathways to complement any knowledge gained from the simulated exercise.

There are several limitations identified with our study. All of the trainees filled out the online survey within a week of completing the simulation session; significantly more trainees reported they were confident and comfortable managing a case of sexual assault. However, when surveyed 8 months later to assess if there had been a translation of perceived competence to improved clinical care, only 61% responded to this follow-up survey. The majority (72%) stated that they were still confident and comfortable in the care of sexual assault patients, especially for obtaining the relevant history (81%) and collecting forensic evidence (72%). However, only 36% could still describe the health consequences of sexual assault, and only 45% said they could still describe the medication treatment options for patients with sexual assault. While the survey response rate limits the interpretation of this potential degradation of the ability to describe the essential components of the medical evaluation for sexual assault, it may be necessary to have refresher opportunities for residents to support their clinical skills in treating patients with sexual assault. Health consequences may need to be further emphasized in the PowerPoint presentation (Appendix F) used during the debriefing. Whether this simulation is also helpful to the future care that patients who have been sexually assaulted receive would also be an optimal goal for such training and should serve as a focus of a future investigation.

We learned several vital lessons while devising and implementing this simulated scenario. The first is that at least two, but not more than four, learners are ideal for this simulation. We found that learners generally divided up the tasks in the care of the patient: One obtained the history, two performed the physical examination, and the other learners assisted with setting up the rape kit and placing the evidence appropriately in it, according to the ED checklist. With more than four learners, the remainder were passively engaged in the simulation and were necessarily bystanders. The second lesson is that ideally, the content expert should not be in the simulation suite with the learners but instead readily available for consultation at the request of the participants and positioned in the observation/viewing room. This allows the learners to feel unencumbered by engaging with the scenario and not looking to the content expert for real-time feedback on any of their actions during the simulation. The third lesson is that clearly labeling each step of the evidence collection with concise directions or tips as to how to best perform that step of the evidence collection process, as we did with our ED checklist, is extremely helpful for learners.

While the above lessons were deemed helpful for implementation of the scenario, they may also limit generalizability. Our intervention took place in a dedicated simulation center with high-fidelity manikins, the ability to view the participants via one-way glass, and a dedicated debriefing room. We feel that this scenario could be adapted to either low-fidelity or in situ simulation, but the effect of this change on learner engagement and retention would need to be further studied.

Conclusion

Emergency medicine trainees who participated in a single-session, high-fidelity simulation of an adolescent with sexual assault subsequently had increased confidence and perceived competence in the medical and forensic evaluation of such patients; however, this perceived competence and confidence diminished in the months after the simulation. Further study to determine if this translates to future optimal care of adolescents with sexual assault is needed.

Appendices

A. Simulator.docx
B. Forensic Evidence Collection Primer.docx
C. Card Layout.docx
D. Sexual Assault Case.docx
E. Critical Actions Checklist.docx
F. Debriefing Presentation.ppt
G. Postsession Survey.docx
H. Follow-up Survey.docx

All appendices are peer reviewed as integral parts of the Original Publication.

Copyright © 2020 Bechtel et al. This is an open-access publication distributed under the terms of the Creative Commons Attribution license.

Kirsten Bechtel, MD: Associate Professor, Departments of Pediatrics and Emergency Medicine, Yale School of Medicine

Ambika Bhatnagar, MBBS: Research Associate, Departments of Pediatrics and Emergency Medicine, Yale School of Medicine

Melissa Joseph, MD: Assistant Professor, Department of Emergency Medicine, Yale School of Medicine

Marc Auerbach, MD: Associate Professor, Departments of Pediatrics and Emergency Medicine, Yale School of Medicine

Disclosures

None to report.

Funding/Support

None to report.

Ethical Approval

Reported as not applicable.

References

1. Davis RC, Auchter B, Howley S, Camp T, Knecht I, Wells W. Increasing the accessibility of sexual assault forensic examinations: evaluation of Texas law SB 1191. *J Forensic Nurs*. 2017;13(4):168-177. https://doi.org/10.1097/JFN.0000000000000169
2. Campbell R, Patterson D, Lichty LF. The effectiveness of sexual assault nurse examiner (SANE) programs: a review of psychological, medical, legal, and community outcomes. *Trauma Violence Abuse*. 2005;6(4):313-329. https://doi.org/10.1177/1524838005280328
3. Crandall CS, Helitzer D. *An Impact Evaluation of a Sexual Assault Nurse Examiner (SANE) Program*. National Criminal Justice Reference Service; 2003.
4. Derhammer F, Lucente V, Reed JF, Young MJ. Using a SANE interdisciplinary approach to care of sexual assault victims. *Jt Comm J Qual Improv*. 2000;26(8):488-496. https://doi.org/10.1016/S1070-3241(00)26041-0
5. Ledray LE, Simmelink K. Efficacy of SANE evidence collection: a Minnesota study. *J Emerg Nurs*. 1997;23(1):75-77. https://doi.org/10.1016/S0099-1767(97)90070-2
6. Lenehan GP. Sexual assault nurse examiners: a SANE way to care for rape victims. *J Emerg Nurs*. 1991;17(1):1-2.
7. Lewis-O'Connor A. The evolution of SANE/SART—are there differences? *J Forensic Nurs*. 2009;5(4):220-227. https://doi.org/10.1111/j.1939-3938.2009.01057.x
8. Sievers V, Murphy S, Miller JJ. Sexual assault evidence collection more accurate when completed by sexual assault nurse examiners: Colorado's experience. *J Emerg Nurs*. 2003;29(6):511-514. https://doi.org/10.1016/S0099-1767(03)00422-7
9. Amey AL, Bishai D. Measuring the quality of medical care for women who experience sexual assault with data from the National Hospital Ambulatory Medical Care Survey. *Ann Emerg Med*. 2002;39(6):631-638. https://doi.org/10.1067/mem.2002.123357
10. Bechtel K, Ryan E, Gallagher D. Impact of sexual assault nurse examiners on the evaluation of sexual assault in a pediatric emergency department. *Pediatr Emerg Care*. 2008;24(7):442-447. https://doi.org/10.1097/PEC.0b013e31817de11d
11. Patel A, Panchal H, Piotrowski ZH, Patel D. Comprehensive medical care for victims of sexual assault: a survey of Illinois hospital emergency departments. *Contraception*. 2008;77(6):426-430. https://doi.org/10.1016/j.contraception.2008.01.018
12. Patel A, Roston A, Tilmon S, et al. Assessing the extent of provision of comprehensive medical care management for female sexual assault patients in US hospital emergency departments. *Int J Gynaecol Obstet*. 2013;123(1):24-28. https://doi.org/10.1016/j.ijgo.2013.04.014
13. Campbell R, Bybee D. Emergency medical services for rape victims: detecting the cracks in service delivery. *Womens Health*. 1997;3(2):75-101.
14. Goyal MK, Mollen CJ, Hayes KL, et al. Enhancing the emergency department approach to pediatric sexual assault care: implementation of a pediatric sexual assault response team program. *Pediatr Emerg Care*. 2013;29(9):969-973. https://doi.org/10.1097/PEC.0b013e3182a21a0d
15. Sampsel K, Szobota L, Joyce D, Graham K, Pickett W. The impact of a sexual assault/domestic violence program on ED care. *J Emerg Nurs*. 2009;35(4):282-289. https://doi.org/10.1016/j.jen.2008.07.014
16. Hornor G, Thackeray J, Scribano P, Curran S, Benzinger E. Pediatric sexual assault nurse examiner care: trace forensic evidence, ano-genital injury, and judicial outcomes. *J Forensic Nurs*. 2012;8(3):105-111. https://doi.org/10.1111/j.1939-3938.2011.01131.x
17. Ferguson C, Faugno D. The SAFE CARE model: maintaining competency in sexual assault examinations utilizing patient simulation methods. *J Forensic Nurs*. 2009;5(2):109-114. https://doi.org/10.1111/j.1939-3938.2009.01042.x
18. Schilling S, Samuels-Kalow M, Gerber JS, Scribano PV, French B, Wood JN. Testing and treatment after adolescent sexual assault in pediatric emergency departments. *Pediatrics*. 2015;136(6):e1495-e1503. https://doi.org/10.1542/peds.2015-2093
19. McGrath ME, Bettacchi A, Duffy SJ, Peipert JF, Becker BM, St. Angelo L. Violence against women: provider barriers to intervention in emergency departments. *Acad Emerg Med*. 1997;4(4):297-300. https://doi.org/10.1111/j.1553-2712.1997.tb03552.x
20. Black MC, Basile KC, Breiding MJ, et al. *The National Intimate Partner and Sexual Violence Survey (NISVS): 2010 Summary Report*. National Center for Injury Prevention and Control, Centers for Disease Control and Prevention; 2011.

Copyright © 2020 Bechtel et al. This is an open-access publication distributed under the terms of the Creative Commons Attribution license.

21. Sheets LK, Bretl D. The SA Pocket Tool. *MedEdPORTAL*. 2012;8:9165. https://doi.org/10.15766/mep_2374-8265.9165
22. Siegel M, Chen Gonzalez E, Wijesekera O, et al. On-the-go training: downloadable modules to train medical students to care for adult female sexual assault survivors. *MedEdPORTAL*. 2017; 13:10656. https://doi.org/10.15766/mep_2374-8265.10656
23. Fitzpatrick M, Ta A, Lenchus J, Arheart KL, Rosen LF, Birnbach DJ. Sexual assault forensic examiners' training and assessment using simulation technology. *J Emerg Nurs*. 2012;38(1):85-90.e86. https://doi.org/10.1016/j.jen.2010.10.002
24. McLaughlin SA, Monahan C, Doezema D, Crandall C. Implementation and evaluation of a training program for the management of sexual assault in the emergency department. *Ann Emerg Med*. 2007;49(4):489-494. https://doi.org/10.1016/j.annemergmed.2006.07.933
25. Auten JD, Ross EM, French MA, et al. Low-fidelity hybrid sexual assault simulation training's effect on the comfort and competency of resident physicians. *J Emerg Med*. 2015;48(3): 344-350. https://doi.org/10.1016/j.jemermed.2014.09.032
26. Sande MK, Broderick KB, Moreira ME, Bender B, Hopkins E, Buchanan JA. Sexual assault training in emergency medicine residencies: a survey of program directors. *West J Emerg Med*. 2013;14(5):461-466. https://doi.org/10.5811/westjem.2013.2.12201
27. Crawford-Jakubiak JE, Alderman EM, Leventhal JM; AAP Committee on Child Abuse and Neglect; AAP Committee on Adolescence. Care of the adolescent after an acute sexual assault. *Pediatrics*. 2017;139(3):e20164243. https://doi.org/10.1542/peds.2016-4243

Received: May 20, 2019
Accepted: January 25, 2020
Published: August 26, 2020

Copyright © 2020 Bechtel et al. This is an open-access publication distributed under the terms of the Creative Commons Attribution license.
