# Supplementary figures and images for: Sexual Assault in an Adolescent Female: A Pediatric Simulation Case for Emergency Medicine Providers
Source: MedEdPORTAL. 2020 Aug 26;16:10942. doi: 10.15766/mep_2374-8265.10942 (PMC7449576; doi:10.15766/mep_2374-8265.10942)

Appendix A: SimMan with Pelvic Anatomy Attachment


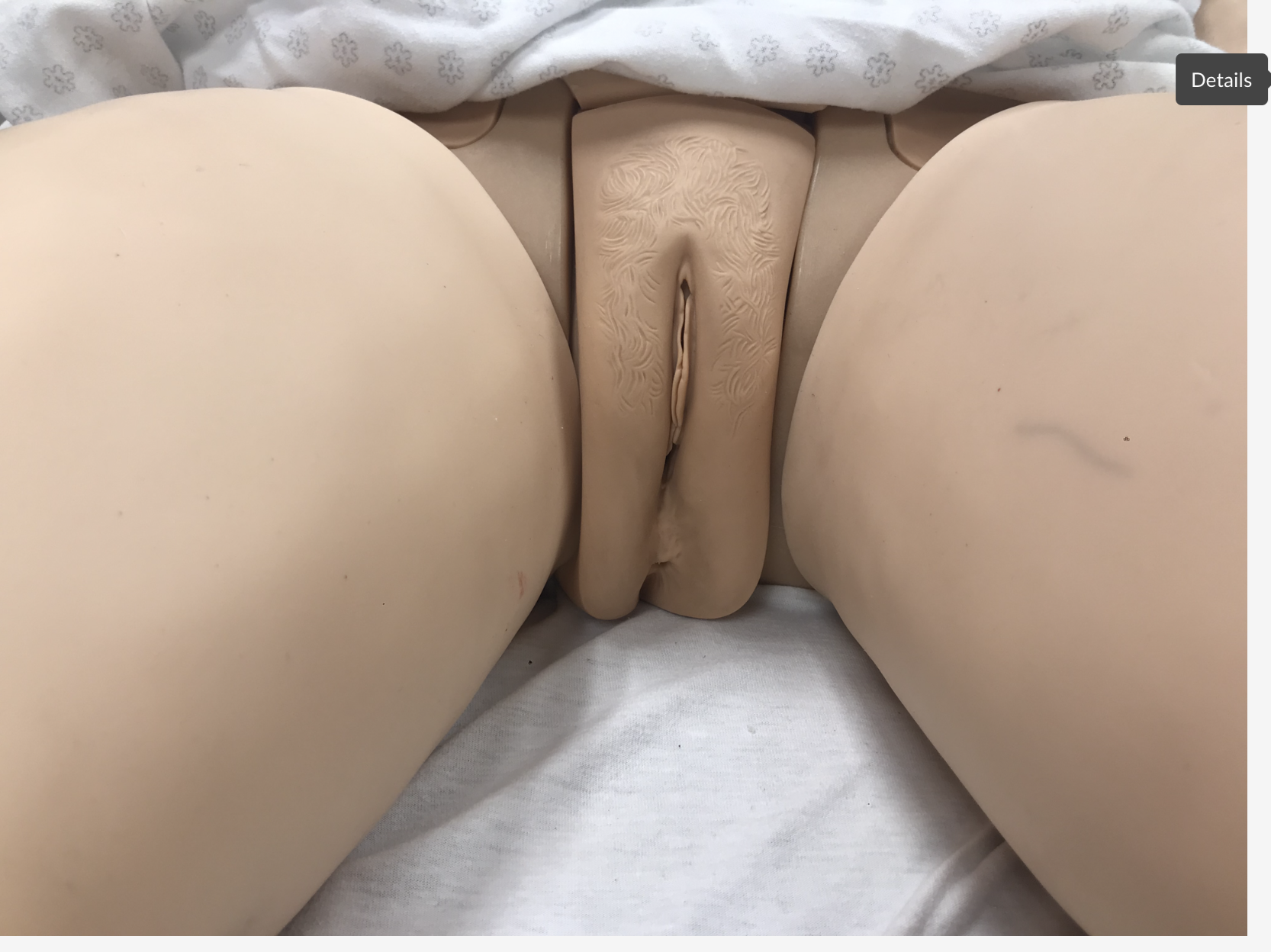

Supplement: Supplementary file 1 — Simulator.docxForensic Evidence Collection Primer.docxCard Layout.docxSexual Assault Case.docxCritical Actions Checklist.docxDebriefing Presentation.pptPostsession Survey.docxFollow-up Survey.docx [file mep_2374-8265.10942-s001.zip › A. Simulator.docx]

Appendix C: Cognitive aids for each step of the forensic evidence collection process


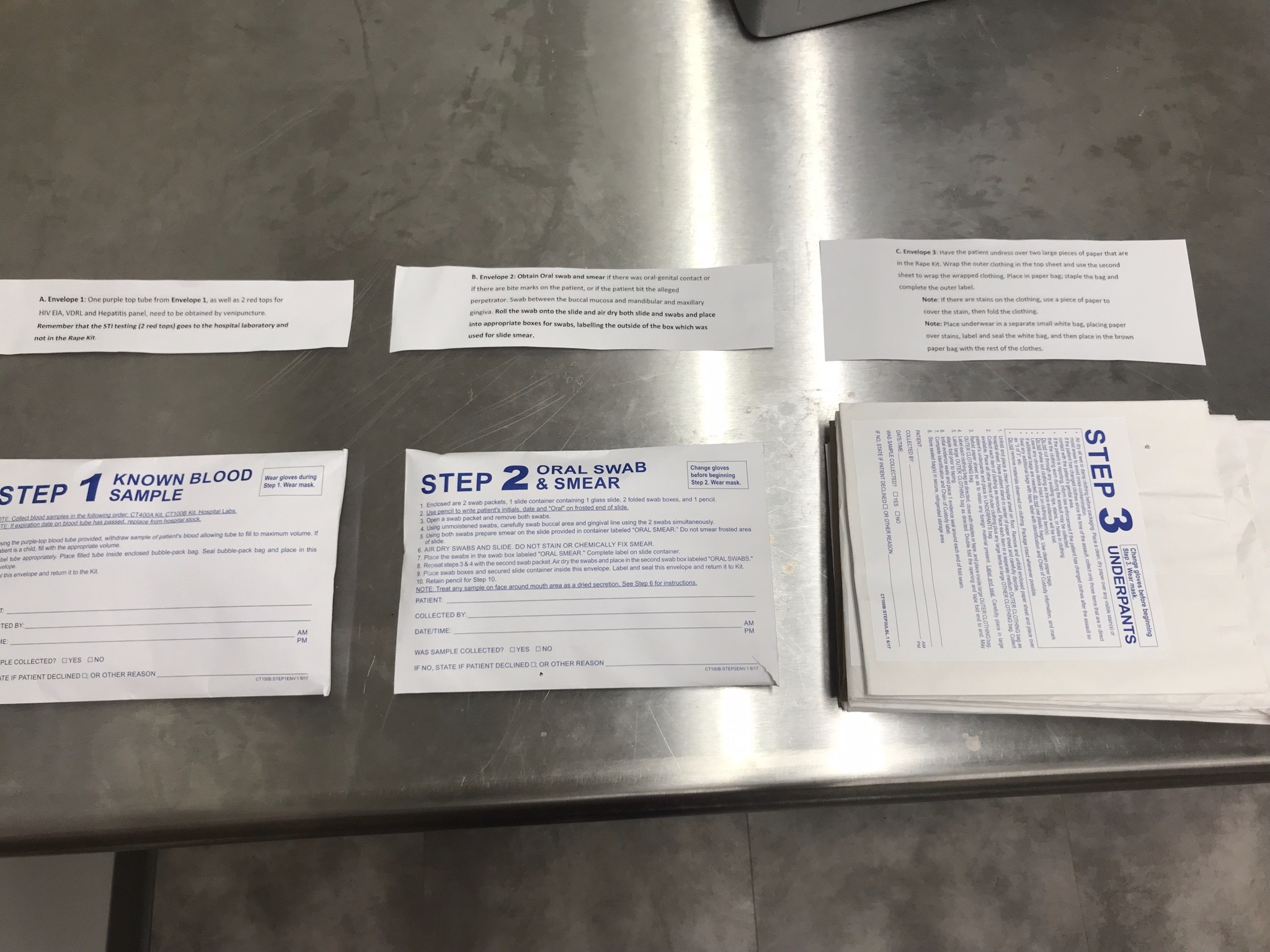

Supplement: Supplementary file 1 — Simulator.docxForensic Evidence Collection Primer.docxCard Layout.docxSexual Assault Case.docxCritical Actions Checklist.docxDebriefing Presentation.pptPostsession Survey.docxFollow-up Survey.docx [file mep_2374-8265.10942-s001.zip › C. Card Layout.docx]
